# Elderly Patient With Bilateral Central Serous Chorioretinopathy Misdiagnosed as Bilateral Wet Age-Related Macular Degeneration: A Case Report

**DOI:** 10.7759/cureus.64210

**Published:** 2024-07-10

**Authors:** Amal Alomari, Sara Issa, Mohammad Abusamak

**Affiliations:** 1 Department of Special Surgery, Al-Balqa Applied University, As-Salt, JOR; 2 Department of Ophthalmology, Amman Eye Clinic, Amman, JOR; 3 Department of Ophthalmology, Al-Balqa Applied University, As-Salt, JOR

**Keywords:** subretinal fluid, double-layer sign, choroidal neovascularisation, wet age related macular degeneration, central serous chorioretinopathy

## Abstract

This report presents a unique case of a 77-year-old diabetic male patient with bilateral central serous chorioretinopathy (CSCR), who was receiving multiple bilateral intravitreal injections for a presumed diagnosis of wet age-related macular degeneration (AMD). The fundus examination did not show any signs of AMD or diabetic retinopathy (DR). The spectral domain optical coherence tomography (OCT) revealed bilateral subretinal fluid. The neovascular membrane was not visible on OCT angiography. Fundus fluorescein angiography (FFA) confirmed the absence of choroidal neovascularization (CNV). Notably, this represents a unique case of an elderly patient with CSCR mimicking occult CNV.

## Introduction

Central serous chorioretinopathy (CSCR) is defined as the detachment of the neurosensory retina caused by the accumulation of fluid from the choriocapillaris in the subretinal space. At times, retinal pigment epithelium (RPE) detachment (PED) may be seen on optical coherence tomography (OCT) in up to 63% of the eyes [1].

The exact underlying pathophysiologic mechanisms remain unclear; nevertheless, contributory factors include stress, heightened cortisol levels, and hypertension [2,3]. Genetic factors were linked to some cases. In a case series, Weenink et al. found that in 27 patients with bilateral CSCR, at least half had one family member with CSCR [4].

CSCR predominantly affects men aged between their 20s and 50s, with a significantly higher incidence rate in males (9.9/100,000) compared to females (1.7/100,000), approximately six times more. Patients often have short- or long-term problems with their central vision and other symptoms such as micropsia, hyperopic or myopic shift, and lessened contrast sensitivity and color saturation [5].

Adrean et al. revealed in a case series that among 522 patients, 11 individuals were misdiagnosed and managed as having wet age-related macular degeneration (AMD), only for a subsequent diagnosis to confirm CSCR as the actual condition [6]. In this paper, we are presenting a case from Jordan with similar findings of bilateral CSCR presenting in an elderly patient and misdiagnosed and managed as wet AMD.

## Case presentation

A 77-year-old male patient from Jordan with type 2 diabetes and hypertension presented to the medical retina clinic complaining of a gradual decrease in vision in his left eye over one year. He has been receiving bilateral multiple intravitreal anti-vascular endothelial growth factor (VEGF) injections (Aflibercept) for a presumed diagnosis of wet AMD in a general ophthalmology clinic, but no significant subjective or objective improvement has been confirmed by OCT macula.

Upon examination, his visual acuity (VA) was 6/6 in his right eye and 6/12 in his left eye. The anterior segment examination revealed moderate nuclear sclerosis in the right eye and pseudophakia in the left eye. Intraocular pressure was 14 mmHg in both eyes. The fundus examination was unremarkable, apart from having a blunt foveal reflex. We observed no macular drusen or intra- or subretinal hemorrhages. We noted no evidence of diabetic retinopathy or maculopathy.

The patient denied having any of the CSCR risk factors, including a recent history of stress or steroid intake. He is a non-smoker with no history of sleep apnea.

**Figure 1:**
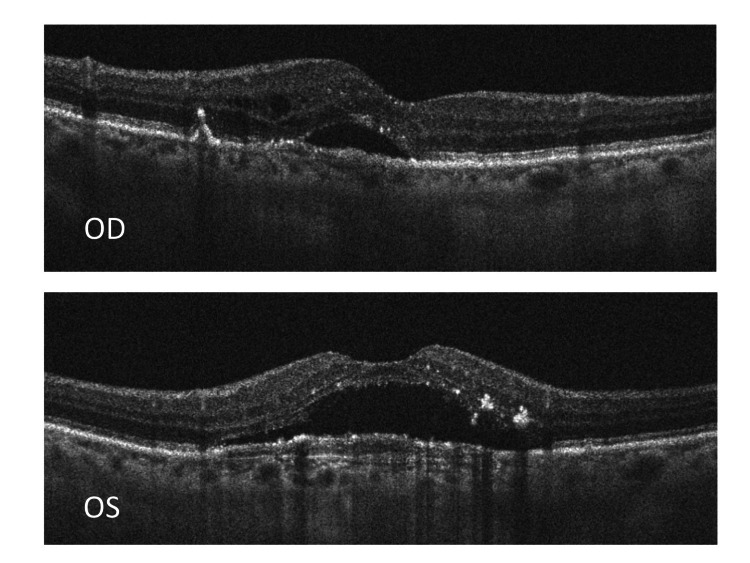
Oculus dexter (OD): OCT of the right eye showing subretinal fluid and multiple small intraretinal cysts with a small pigment epithelial detachment; oculus sinister (OS): OCT of the left eye showing subretinal fluid, double-layer sign, and loss of outer retinal layers; OCT: optical coherence tomography.

The spectral domain macular OCT (Figure 1) showed that there was neurosensory detachment on both sides because of the buildup of subretinal fluid in both eyes. The macular thickness was 358 and 452 µn in the right and left eyes, respectively. Both eyes showed a double-layer sign (DLS) on OCT, and the left eye revealed a hyporeflective gap within the DLS. Sheth et al. described the DLS as the shallow and irregular elevation of the RPE layer from the underlying intact Burch’s membrane [7]. While the right eye OCT (OD) revealed intraretinal cysts, indicating chronicity, the left eye (OS) showed a more significant loss of the outer retinal layers, which denotes a worse prognosis.

**Figure 2:**
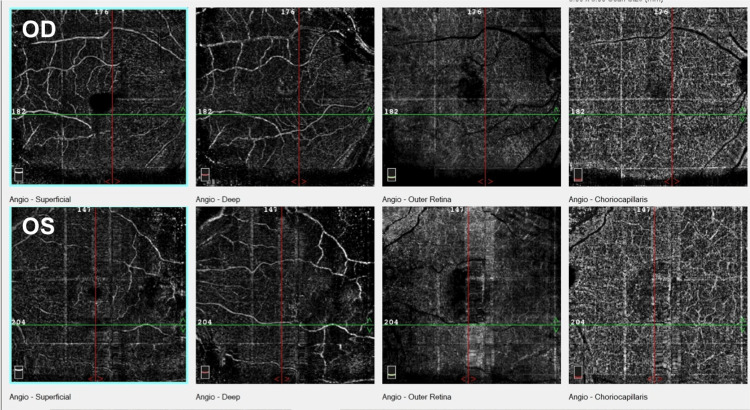
OCT angiography of the right (OD) and left (OS) eyes showing superficial, deep, outer retina, and choriocapillaris layers consecutively from left to right. No evidence of abnormal choroidal vessels was detected in the choriocapillaris layer. A dark area that corresponds to subretinal fluid was seen in the choriocapillaris, more obvious in the left eye. OCT: optical coherence tomography; OD: oculus dexter; OS: oculus sinister

OCT angiography (Figure 2) did not show any neovascular membranes.

**Figure 3:**
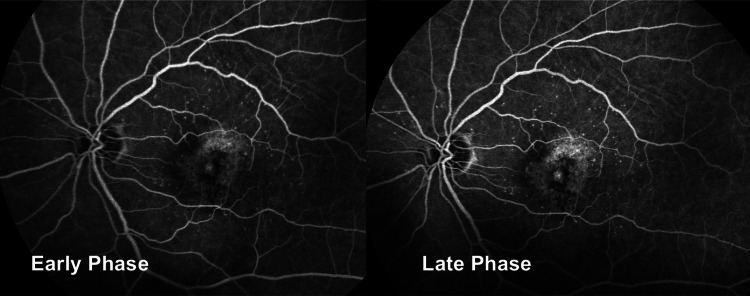
Early phase and late phase showing central window defect with mottled hyper-fluorescence with no leakage observed.

The right-eye fundus fluorescein angiography (FFA) was of poor quality because of cataracts, while the left-eye FFA (Figure 2) showed a central area of early hyper-fluorescence because of a window defect with patches of mottled hyper-fluorescence. Throughout the test period, there was no evidence of any leakage (Figure 3).

## Discussion

In this case report, we present the intriguing case of an older man, initially diagnosed and treated for bilateral wet AMD because of the presence of subretinal fluid on an OCT, a common sign of wet AMD in this age group. Upon closer inspection, we suggest an alternative diagnosis of bilateral chronic CSCR, bolstered by the following observations.

First, the patient demonstrated VA that exceeded typical levels seen in chronic wet AMD cases, and he did not report any metamorphopsia. Furthermore, the absence of macular drusen or hemorrhages makes AMD less likely. OCT imaging revealed that chronic subretinal fluid was unresponsive to intravitreal anti-VEGF injections. Both FFA and OCT angiography failed to reveal any signs of choroidal neovascularization (CNV), further supporting the diagnosis of chronic CSCR over wet AMD.

Adrian et al., in a case series reviewing 522 elderly patients with initial diagnoses of wet AMD, reported findings consistent with this notion. Interestingly, eleven patients met the diagnostic criteria for CSCR. On spectral domain-OCT (SD-OCT) scans, all these individuals showed subretinal fluid, and 66.7% showed DLS. No signs of intra- or subretinal hemorrhage were detected. All these patients had excellent initial vision, and there was minimal to no response to anti-VEGF therapy. Therefore, they favored the diagnosis of CSCR for this specific cohort [6].

In 1992, Schatz et al. documented the appearance of CSCR in the eyes of 13 patients aged 60 years or older, a condition typically observed in adults. Interestingly, none of the patients in this series exhibited soft drusen, geographic atrophy, or subretinal neovascularization, which are hallmark signs of AMD or other retinal or macular diseases. They concluded that older individuals might mistakenly diagnose CSCR as macular degeneration with subretinal neovascularization [8].

## Conclusions

In elderly patients, CSCR can often resemble an occult nAMD. When nAMD cannot be ruled out, a trial of intravitreal anti-VEGF is recommended. Our paper presents the case of an elderly patient initially diagnosed with nAMD, which was later found to be more consistent with CSCR. In elderly individuals, CSCR should be considered when patients show poor or no response to anti-VEGF agents, with intervals of resolution of subretinal fluid not attributable to anti-VEGF therapy. Historical factors, initial excellent vision, and examination findings, such as the absence of retinal hemorrhage, can aid in establishing the diagnosis. Typically, the condition improves with monitoring, but vigilant follow-up is necessary because of the potential for conversion to choroidal neovascularization (CNVM).
